# Structure of Λ(δλλ)-[Co(en)_3_]I_3_(I)_2_

**DOI:** 10.1107/S2056989021002826

**Published:** 2021-03-31

**Authors:** Megan R. Kollitz, Allen G. Oliver, A. Graham Lappin

**Affiliations:** a University of Notre Dame, Department of Chemistry and Biochemistry, 251 Nieuwland Science Hall, Notre Dame IN 46556, USA

**Keywords:** crystal structure, cobalt coordination, chiral coordination

## Abstract

The structure of tris­(ethane-1,2-di­amine-κ^2^ *N,N*’)cobalt(III) bis­(iodide) triiodide, [Co(C_2_H_8_N_2_)_3_]I_3_(I)_2_, at 120 K has ortho­rhom­bic (*P*2_1_2_1_2_1_) symmetry. The di­amine nitro­gen atoms form N—H⋯I hydrogen bonds throughout the lattice, resulting in a three-dimensional network, which involves the iodide and all atoms in the triiodide anions.

## Abstract 2

The structure, coordination geometry and extended hydrogen-bonded network of tris­(ethane-1,2-di­amine-κ^2^ *N*,*N*′)cobalt(III) bis­(iodide) triiodide is discussed.

## Chemical context   

Significant information on the hydrogen bonding and other inter­actions that contribute to the chiral discriminations between metal-ion complexes has been obtained from the crystal structures of compounds containing a chiral complex cation and a chiral complex anion (Warren *et al.*, 1994 ▸; Marusak & Lappin, 1989 ▸). For example, a comparison of the compounds Λ-[Co(en)_3_]Δ-[Co(en)(ox)_2_]I_2_·3H_2_O and Δ-[Co(en)_3_]Δ-[Co(en)(ox)_2_]I_2_·H_2_O reveals the importance of different helicities projected along the *C*_3_ and *C*_2_ axes of [Co(en)_3_]^3+^ in discriminating with the pseudo-*C*_3_ face of the Δ-[Co(en)(ox)_2_]^−^ anion (Lappin *et al.*, 1993 ▸).
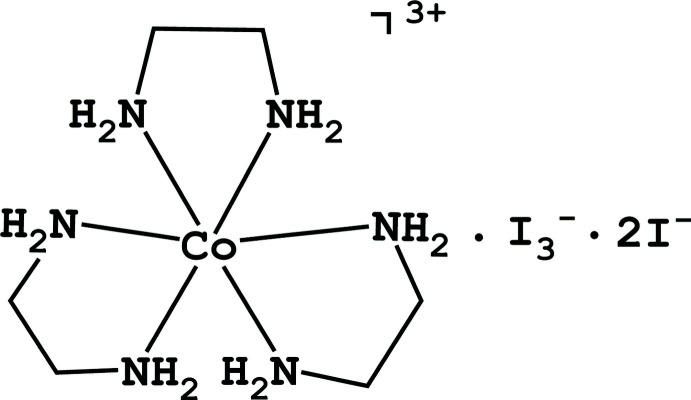


As part of a study involving potential effects of non-chiral counter-ions, an attempt was made to grow crystals with [Co(en)_3_]I_3_ and Na[Co(edta)]. However, in the presence of I^−^, the mildly oxidizing [Co(edta)]^−^ was reduced and an unexpected product, [Co(en)_3_]I_3_(I)_2_ was obtained. The structure of the corresponding cobalt(II) complex, [Co(en)_3_]I_3_I, has been reported (Du *et al.*, 2007 ▸). The larger cobalt(II) complex supports an *lel*_3_ geometry of the bidentate ligands around the cobalt center. The Co—N bond distances in [Co(en)_3_]^2+^ average 2.28 Å, significantly longer than the 1.97 Å average in [Co(en)_3_]^3+^ and consistent with the sluggish redox exchange between the complexes (Jolley *et al.*, 1990 ▸). In [Co(en)_3_]I_3_I, the I^−^ ions are located along the quasi-*C*_2_ axis of the [Co(en)_3_]^2+^ complex ion with close hydrogen-bond contacts from N—H protons of 2.91 Å. The terminal iodine atoms of the I_3_^−^ ions likewise form hydrogen bonds with N—H protons at 2.93 Å, resulting in an alternating chain of linear I_3_^−^ ions at 90° to one another down the *c*-axis direction.

## Structural commentary   

**Figure 1:**
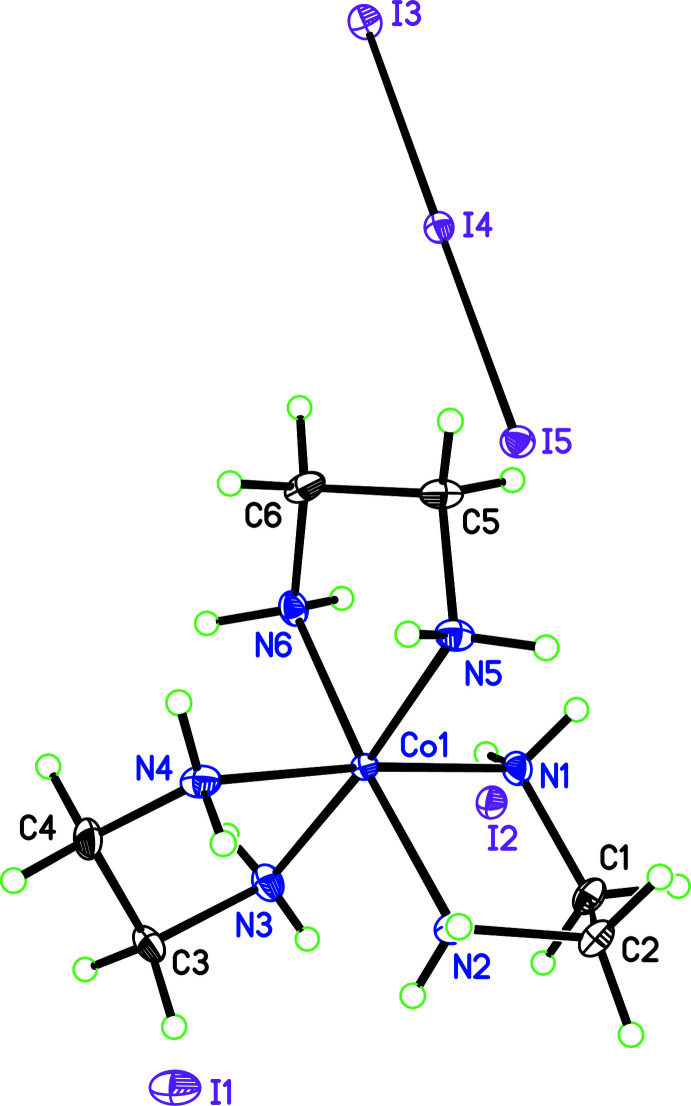
Labeling scheme for [Co(en)_3_]I_3_(I)_2_ with ellipsoids at the 50% probability level. Hydrogen atoms depicted as spheres of an arbitrary radius.

The complex, [Co(en)_3_](I_3_)(I)_2_ crystallizes as dark-red, rod-like crystals. The asymmetric unit of the primitive, acentric, ortho­rhom­bic space group *P*2_1_2_1_2_1_ consists of one [Co(en)_3_]^3+^ cation, two iodide anions and a triiodide anion (Fig. 1 ▸). The correct enanti­omorph of the space group was determined by comparison of intensities of Friedel pairs of reflections, yielding a Flack *x* parameter of 0.017 (9) (Parsons *et al.*, 2013 ▸) and a Hooft *y* parameter of 0.006 (8) (Hooft *et al.*, 2008 ▸). Values close to zero indicate the correct enanti­omorph of the space group. This determination allows an accurate assessment of the configuration of the cobalt cation.

The cobalt center is located in a slightly distorted octa­hedral environment by the nitro­gen atoms of three ethyl­ene di­amine ligands (see Table 1 ▸ for details). The ligands adopt a Λ(δλλ) *lel ob ob* (*lelob_2_*) geometry about the cobalt center, Fig. 1 ▸. Bond distances and angles within the mol­ecules are unexceptional.

**Figure 2:**
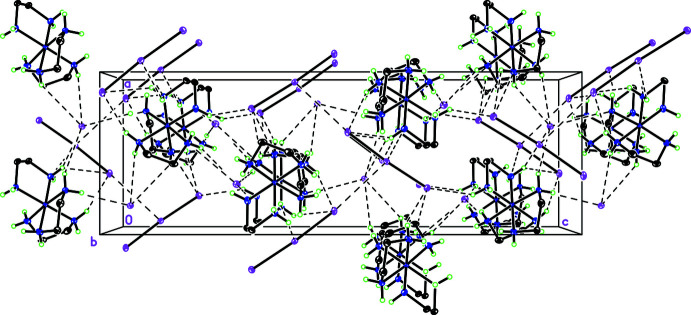
View down the *b* axis of [Co(en)_3_]I_3_(I)_2_ showing the herringbone pattern, with ellipsoids at the 50% probability level.

The amine hydrogen atoms were initially located from a difference-Fourier map and were refined freely. All of the amine hydrogen atoms are involved in hydrogen bonds to nearby iodine/triiodide moieties, Fig. 2 ▸. This inter­connectivity results in a three-dimensional hydrogen-bonded network throughout the entire structure.

## Supra­molecular features   

The iodide ion I^−^(1) is hydrogen bonded to N—H protons from N4 on one [Co(en)_3_]^3+^ ion at 2.77 Å, bridging to N—H protons on N4 and N5 from the two ligands with a λ-configuration on an adjacent cation with distances of 2.90 (5) and 2.95 (5) Å (Fig. 2 ▸, Table 2 ▸). The pairwise inter­actions create a hydrogen-bonded chain along the crystallographic *a*-axis direction, forming a layer with the complex cations separated by channels formed by I_3_^−^ ions in an alternating herringbone pattern punctuated by I2^−^ ions. The iodide I2^−^ forms a hydrogen-bonded network bridging the layers with N—H protons from three separate cations at 2.79 (5), 2.80 (5) and 2.83 (5) Å. The I_3_^−^ ion has a close N—H contact with N6 at 2.89 (5) Å.

## Database survey   

A survey of Co(en)_3_ coupled with iodine reveals 23 structures in the Cambridge Structural Database (CSD v5.42, November 2020; Groom *et al.*, 2016 ▸). Predominantly these are Co(III) complexes. There are three reports of Co(en)_3_I_3_ (EDANEC, Matsuki *et al.*, 2001 ▸; ENCOIH, Whuler *et al.*, 1980 ▸; FIXLAI, Grant *et al.*, 2019 ▸). EDANEC and FIXLAI are structural analyses of the Λ- and Δ-isomers, respectively. The structure determination by Whuler *et al.* is of the racemic cation species. A mixed Cl/I species was reported by Huang and co-workers (FAXMEX, Zhang *et al.*, 2005 ▸). All of these reports also contain water of crystallization. There is one report of Co(en)_3_ that has both an iodide and a triodide pair of counter-ions that crystallizes in the tetra­gonal space group *I* 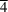
2d (HIQYUC, Du *et al.*, 2007 ▸). However, that report is of the Co^II^ complex, Co(en)_3_(I_3_)I.

## Synthesis and crystallization   

Crystals were obtained from an attempt to co-crystallize optically active [Co(en)_3_]^3+^ and the mildly oxidizing [Co(edta)]^−^ from Λ-[Co(en)_3_]I_3_ and Na[Co(edta)]. After storage at 283 K for two weeks, the deep-purple coloration of the [Co(edta)]^−^ ion had disappeared and dark-red well-formed crystals were recovered.

## Refinement   

Crystal data, data collection and structure refinement details are summarized in Table 3 ▸. The structure was solved by dual-space methods (Sheldrick, 2015*a* ▸) and refined routinely (Sheldrick, 2015*b* ▸). Amine hydrogen atoms were refined freely and methyl­ene hydrogen atoms were refined as riding on the carbon to which they are bonded with C—H = 0.99 Å and *U*_iso_(H) = 1.2*U*_eq_(C).

## Supplementary Material

Crystal structure: contains datablock(s) I. DOI: 10.1107/S2056989021002826/dj2023sup1.cif

Structure factors: contains datablock(s) I. DOI: 10.1107/S2056989021002826/dj2023Isup2.hkl

CCDC reference: 2070495

Additional supporting information:  crystallographic information; 3D view; checkCIF report

## Figures and Tables

**Table 1:** Selected geometric parameters (Å, °)

Co1—N1	1.964 (3)	Co1—N3	1.968 (3)
Co1—N5	1.967 (3)	Co1—N6	1.969 (3)
Co1—N4	1.968 (3)	Co1—N2	1.982 (3)
			
N1—Co1—N5	91.86 (14)	N4—Co1—N6	91.39 (13)
N1—Co1—N4	175.38 (14)	N3—Co1—N6	91.83 (13)
N5—Co1—N4	91.64 (14)	N1—Co1—N2	85.20 (13)
N1—Co1—N3	91.25 (13)	N5—Co1—N2	91.96 (14)
N5—Co1—N3	175.65 (14)	N4—Co1—N2	91.68 (13)
N4—Co1—N3	85.42 (14)	N3—Co1—N2	91.34 (15)
N1—Co1—N6	91.91 (13)	N6—Co1—N2	175.76 (13)
N5—Co1—N6	85.02 (13)		

**Table 2:** Hydrogen-bond geometry (Å, °)

*D*—H⋯*A*	*D*—H	H⋯*A*	*D*⋯*A*	*D*—H⋯*A*
N1—H1*C*⋯I2	0.87 (5)	2.83 (5)	3.598 (3)	149 (4)
N1—H1*D*⋯I2^i^	0.87 (5)	2.79 (5)	3.616 (3)	158 (4)
N2—H2*C*⋯I3^ii^	0.89 (5)	3.04 (5)	3.823 (3)	149 (4)
N2—H2*D*⋯I1^iii^	0.80 (5)	3.02 (5)	3.756 (4)	154 (4)
N3—H3*C*⋯I2^iv^	0.97 (5)	3.25 (5)	4.111 (3)	149 (4)
N3—H3*C*⋯I5^iv^	0.97 (5)	3.08 (5)	3.586 (3)	114 (3)
N3—H3*D*⋯I1^iii^	0.76 (5)	3.18 (5)	3.805 (4)	142 (5)
N4—H4*C*⋯I1	0.92 (5)	2.77 (5)	3.630 (3)	155 (4)
N4—H4*D*⋯I1^v^	0.90 (5)	2.90 (5)	3.715 (3)	152 (4)
N5—H5*C*⋯I1^v^	0.89 (5)	2.95 (5)	3.765 (3)	154 (4)
N5—H5*C*⋯I3^ii^	0.89 (5)	3.25 (5)	3.639 (3)	109 (3)
N5—H5*D*⋯I4^ii^	0.89 (5)	3.05 (5)	3.611 (3)	123 (4)
N6—H6*C*⋯I5	0.91 (5)	2.89 (5)	3.674 (3)	145 (4)
N6—H6*D*⋯I2^iv^	0.95 (5)	2.80 (5)	3.684 (3)	157 (4)

Symmetry codes: (i) -x+1, y+{\script{1\over 2}}, -z+{\script{1\over 2}}; (ii) -x, y+{\script{1\over 2}}, -z+{\script{1\over 2}}; (iii) x+{\script{1\over 2}}, -y+{\script{1\over 2}}, -z+1; (iv) -x+1, y-{\script{1\over 2}}, -z+{\script{1\over 2}}; (v) x-{\script{1\over 2}}, -y+{\script{1\over 2}}, -z+1.

**Table 3:** Experimental details

Crystal data	
Chemical formula	[Co(C_2_H_8_N_2_)_3_]I_3_(I)_2_
*M*_r_	873.74
Crystal system, space group	Orthorhombic, *P*2_1_2_1_2_1_
Temperature (K)	120
*a*, *b*, *c* (Å)	8.7508 (12), 8.8333 (12), 25.982 (4)
*V* (Å^3^)	2008.4 (5)
*Z*	4
Radiation type	Mo *K*α
μ (mm^−1^)	8.54
Crystal size (mm)	0.28 × 0.15 × 0.10

Data collection	
Diffractometer	Bruker Kappa X8 APEXII
Absorption correction	Numerical (*SADABS*; Krause *et al.*, 2015 ▸)
*T*_min_, *T*_max_	0.603, 1.000
No. of measured, independent and observed [*I* > 2σ(*I*)] reflections	35617, 5024, 5023
*R*_int_	0.022
(sin θ/λ)_max_ (Å^−1^)	0.668

Refinement	
*R*[*F* ^2^ > 2σ(*F* ^2^)], *wR*(*F* ^2^), *S*	0.012, 0.028, 1.33
No. of reflections	5024
No. of parameters	199
H-atom treatment	H atoms treated by a mixture of independent and constrained refinement
Δρ_max_, Δρ_min_ (e Å^−3^)	0.39, −0.66
Absolute structure	Flack *x* determined using 2136 quotients [(*I* ^+^)−(*I* ^−^)]/[(*I* ^+^)+(*I* ^−^)] (Parsons *et al.*, 2013 ▸).
Absolute structure parameter	0.017 (9)

Computer programs: *APEX3* and *SAINT* (Bruker, 2015 ▸), *SHELXT2014/5* (Sheldrick, 2015*a* ▸), *SHELXL2018/3* (Sheldrick, 2015*b* ▸), *Mercury* (Macrae *et al.*, 2020 ▸), *CIFTAB* (Sheldrick, 2008 ▸) and *publCIF* (Westrip, 2010 ▸).

## supplementary crystallographic information

### Crystal data

**Table:**

[Co(C_2_H_8_N_2_)_3_]I_3_(I)_2_	*D*_x_ = 2.890 Mg m^−^^3^
*M**_r_* = 873.74	Mo *K*α radiation, λ = 0.71073 Å
Orthorhombic, *P*2_1_2_1_2_1_	Cell parameters from 9680 reflections
*a* = 8.7508 (12) Å	θ = 2.4–28.4°
*b* = 8.8333 (12) Å	µ = 8.54 mm^−^^1^
*c* = 25.982 (4) Å	*T* = 120 K
*V* = 2008.4 (5) Å^3^	Rod, dark red
*Z* = 4	0.28 × 0.15 × 0.10 mm
*F*(000) = 1576	

### Data collection

**Table:**

Bruker Kappa X8 APEXII diffractometer	5024 independent reflections
Radiation source: fine-focus sealed tube	5023 reflections with *I* > 2σ(*I*)
Graphite monochromator	*R*_int_ = 0.022
Detector resolution: 8.33 pixels mm^-1^	θ_max_ = 28.3°, θ_min_ = 1.6°
combination of ω and φ–scans	*h* = −11→11
Absorption correction: numerical (SADABS; Krause *et al*., 2015)	*k* = −11→11
*T*_min_ = 0.603, *T*_max_ = 1.000	*l* = −33→34
35617 measured reflections	

### Refinement

**Table:**

Refinement on *F*^2^	Secondary atom site location: difference Fourier map
Least-squares matrix: full	Hydrogen site location: mixed
*R*[*F*^2^ > 2σ(*F*^2^)] = 0.012	H atoms treated by a mixture of independent and constrained refinement
*wR*(*F*^2^) = 0.028	*w* = 1/[σ^2^(*F*_o_^2^) + (0.0073*P*)^2^ + 1.9769*P*] where *P* = (*F*_o_^2^ + 2*F*_c_^2^)/3
*S* = 1.33	(Δ/σ)_max_ = 0.001
5024 reflections	Δρ_max_ = 0.39 e Å^−^^3^
199 parameters	Δρ_min_ = −0.66 e Å^−^^3^
0 restraints	Absolute structure: Flack *x* determined using 2136 quotients [(*I*^+^)-(*I*^-^)]/[(*I*^+^)+(*I*^-^)] (Parsons *et al*., 2013)
Primary atom site location: dual	Absolute structure parameter: 0.017 (9)

### Special details

**Table:**

Geometry. All esds (except the esd in the dihedral angle between two l.s. planes) are estimated using the full covariance matrix. The cell esds are taken into account individually in the estimation of esds in distances, angles and torsion angles; correlations between esds in cell parameters are only used when they are defined by crystal symmetry. An approximate (isotropic) treatment of cell esds is used for estimating esds involving l.s. planes.

### Fractional atomic coordinates and isotropic or equivalent isotropic displacement parameters (Å^2^)

**Table:**

	*x*	*y*	*z*	*U*_iso_*/*U*_eq_	
I1	0.32971 (3)	0.22384 (3)	0.55282 (2)	0.01429 (5)	
I2	0.69407 (3)	0.30959 (2)	0.22059 (2)	0.01325 (5)	
I3	−0.13267 (3)	0.17300 (3)	0.01437 (2)	0.01367 (5)	
I4	0.05242 (3)	0.28734 (2)	0.09775 (2)	0.01107 (5)	
I5	0.22245 (3)	0.40722 (3)	0.18675 (2)	0.01307 (5)	
Co1	0.32630 (5)	0.30665 (5)	0.36719 (2)	0.00693 (8)	
N1	0.4364 (4)	0.4391 (3)	0.31911 (13)	0.0111 (6)	
H1C	0.466 (6)	0.383 (5)	0.2936 (18)	0.013*	
H1D	0.380 (6)	0.513 (5)	0.3077 (18)	0.013*	
N2	0.4337 (4)	0.4219 (4)	0.42174 (12)	0.0119 (6)	
H2C	0.374 (6)	0.453 (5)	0.4473 (19)	0.014*	
H2D	0.500 (6)	0.370 (5)	0.4332 (19)	0.014*	
N3	0.4917 (4)	0.1557 (3)	0.36466 (13)	0.0121 (6)	
H3C	0.484 (6)	0.087 (5)	0.3358 (18)	0.015*	
H3D	0.571 (6)	0.189 (6)	0.3664 (19)	0.015*	
N4	0.2318 (4)	0.1713 (4)	0.41825 (12)	0.0122 (6)	
H4C	0.248 (5)	0.217 (5)	0.4497 (19)	0.015*	
H4D	0.129 (6)	0.168 (5)	0.4168 (18)	0.015*	
N5	0.1500 (4)	0.4446 (3)	0.36922 (12)	0.0114 (6)	
H5C	0.094 (6)	0.419 (5)	0.3963 (19)	0.014*	
H5D	0.173 (6)	0.543 (5)	0.3687 (18)	0.014*	
N6	0.2158 (4)	0.2066 (3)	0.31060 (12)	0.0109 (5)	
H6C	0.261 (5)	0.236 (5)	0.2807 (19)	0.013*	
H6D	0.227 (5)	0.100 (5)	0.3128 (18)	0.013*	
C1	0.5641 (4)	0.5172 (4)	0.34653 (16)	0.0154 (7)	
H1A	0.595861	0.608878	0.327383	0.018*	
H1B	0.653317	0.448913	0.349678	0.018*	
C2	0.5050 (5)	0.5596 (4)	0.39899 (16)	0.0158 (7)	
H2A	0.589927	0.595429	0.421034	0.019*	
H2B	0.428560	0.641804	0.396078	0.019*	
C3	0.4761 (4)	0.0485 (4)	0.40885 (15)	0.0129 (7)	
H3A	0.521899	0.092664	0.440276	0.016*	
H3B	0.528980	−0.047957	0.401093	0.016*	
C4	0.3078 (5)	0.0210 (4)	0.41678 (15)	0.0146 (7)	
H4A	0.266124	−0.040706	0.388242	0.018*	
H4B	0.290451	−0.033783	0.449508	0.018*	
C5	0.0500 (4)	0.4213 (4)	0.32313 (15)	0.0135 (7)	
H5A	0.089273	0.479960	0.293487	0.016*	
H5B	−0.055557	0.455093	0.330595	0.016*	
C6	0.0521 (4)	0.2540 (4)	0.31111 (15)	0.0135 (7)	
H6A	−0.005054	0.196883	0.337647	0.016*	
H6B	0.004415	0.234598	0.277189	0.016*	

### Atomic displacement parameters (Å^2^)

**Table:**

	*U*^11^	*U*^22^	*U*^33^	*U*^12^	*U*^13^	*U*^23^
I1	0.00933 (10)	0.02215 (11)	0.01140 (10)	0.00063 (9)	−0.00073 (9)	−0.00018 (9)
I2	0.01308 (11)	0.01112 (9)	0.01555 (11)	−0.00011 (8)	−0.00068 (9)	−0.00056 (8)
I3	0.01745 (12)	0.01416 (10)	0.00939 (10)	0.00078 (8)	0.00012 (9)	−0.00136 (8)
I4	0.01061 (10)	0.01122 (9)	0.01138 (10)	0.00010 (8)	0.00211 (8)	0.00068 (8)
I5	0.01294 (11)	0.01450 (10)	0.01176 (11)	−0.00060 (8)	−0.00106 (9)	0.00081 (8)
Co1	0.0067 (2)	0.00784 (19)	0.00628 (19)	−0.00032 (16)	0.00029 (17)	0.00003 (15)
N1	0.0095 (14)	0.0099 (13)	0.0139 (15)	−0.0003 (11)	0.0011 (13)	0.0024 (11)
N2	0.0109 (15)	0.0144 (14)	0.0104 (14)	−0.0011 (12)	−0.0008 (12)	−0.0011 (11)
N3	0.0116 (15)	0.0110 (13)	0.0138 (15)	0.0019 (11)	0.0024 (13)	0.0024 (11)
N4	0.0083 (14)	0.0189 (14)	0.0095 (14)	−0.0023 (12)	0.0009 (12)	0.0023 (12)
N5	0.0108 (15)	0.0130 (14)	0.0104 (14)	0.0028 (11)	−0.0015 (12)	−0.0024 (11)
N6	0.0156 (14)	0.0080 (12)	0.0091 (13)	0.0005 (11)	−0.0008 (12)	−0.0004 (10)
C1	0.0100 (17)	0.0149 (16)	0.0212 (19)	−0.0073 (14)	−0.0008 (16)	0.0043 (14)
C2	0.0150 (18)	0.0125 (16)	0.020 (2)	−0.0049 (14)	−0.0044 (16)	−0.0013 (14)
C3	0.0147 (18)	0.0117 (15)	0.0124 (17)	0.0021 (13)	0.0000 (14)	0.0048 (13)
C4	0.0187 (19)	0.0112 (14)	0.0140 (17)	−0.0041 (14)	−0.0003 (15)	0.0048 (12)
C5	0.0103 (16)	0.0175 (16)	0.0127 (17)	0.0016 (13)	−0.0033 (14)	−0.0019 (13)
C6	0.0100 (16)	0.0178 (16)	0.0126 (16)	−0.0042 (13)	−0.0018 (14)	−0.0011 (13)

### Geometric parameters (Å, º)

**Table:**

I3—I4	2.8875 (4)	N5—H5C	0.89 (5)
I4—I5	2.9464 (4)	N5—H5D	0.89 (5)
Co1—N1	1.964 (3)	N6—C6	1.493 (5)
Co1—N5	1.967 (3)	N6—H6C	0.91 (5)
Co1—N4	1.968 (3)	N6—H6D	0.95 (5)
Co1—N3	1.968 (3)	C1—C2	1.505 (6)
Co1—N6	1.969 (3)	C1—H1A	0.9900
Co1—N2	1.982 (3)	C1—H1B	0.9900
N1—C1	1.494 (5)	C2—H2A	0.9900
N1—H1C	0.87 (5)	C2—H2B	0.9900
N1—H1D	0.87 (5)	C3—C4	1.507 (5)
N2—C2	1.490 (5)	C3—H3A	0.9900
N2—H2C	0.89 (5)	C3—H3B	0.9900
N2—H2D	0.80 (5)	C4—H4A	0.9900
N3—C3	1.495 (5)	C4—H4B	0.9900
N3—H3C	0.97 (5)	C5—C6	1.511 (5)
N3—H3D	0.76 (5)	C5—H5A	0.9900
N4—C4	1.486 (5)	C5—H5B	0.9900
N4—H4C	0.92 (5)	C6—H6A	0.9900
N4—H4D	0.90 (5)	C6—H6B	0.9900
N5—C5	1.498 (5)		
			
I3—I4—I5	176.193 (11)	Co1—N5—H5D	115 (3)
N1—Co1—N5	91.86 (14)	H5C—N5—H5D	113 (4)
N1—Co1—N4	175.38 (14)	C6—N6—Co1	109.8 (2)
N5—Co1—N4	91.64 (14)	C6—N6—H6C	110 (3)
N1—Co1—N3	91.25 (13)	Co1—N6—H6C	107 (3)
N5—Co1—N3	175.65 (14)	C6—N6—H6D	112 (3)
N4—Co1—N3	85.42 (14)	Co1—N6—H6D	111 (3)
N1—Co1—N6	91.91 (13)	H6C—N6—H6D	107 (4)
N5—Co1—N6	85.02 (13)	N1—C1—C2	106.8 (3)
N4—Co1—N6	91.39 (13)	N1—C1—H1A	110.4
N3—Co1—N6	91.83 (13)	C2—C1—H1A	110.4
N1—Co1—N2	85.20 (13)	N1—C1—H1B	110.4
N5—Co1—N2	91.96 (14)	C2—C1—H1B	110.4
N4—Co1—N2	91.68 (13)	H1A—C1—H1B	108.6
N3—Co1—N2	91.34 (15)	N2—C2—C1	107.5 (3)
N6—Co1—N2	175.76 (13)	N2—C2—H2A	110.2
C1—N1—Co1	109.8 (2)	C1—C2—H2A	110.2
C1—N1—H1C	114 (3)	N2—C2—H2B	110.2
Co1—N1—H1C	107 (3)	C1—C2—H2B	110.2
C1—N1—H1D	104 (3)	H2A—C2—H2B	108.5
Co1—N1—H1D	113 (3)	N3—C3—C4	107.2 (3)
H1C—N1—H1D	110 (4)	N3—C3—H3A	110.3
C2—N2—Co1	109.5 (2)	C4—C3—H3A	110.3
C2—N2—H2C	107 (3)	N3—C3—H3B	110.3
Co1—N2—H2C	114 (3)	C4—C3—H3B	110.3
C2—N2—H2D	108 (4)	H3A—C3—H3B	108.5
Co1—N2—H2D	108 (3)	N4—C4—C3	107.3 (3)
H2C—N2—H2D	109 (5)	N4—C4—H4A	110.3
C3—N3—Co1	109.7 (2)	C3—C4—H4A	110.3
C3—N3—H3C	101 (3)	N4—C4—H4B	110.3
Co1—N3—H3C	113 (3)	C3—C4—H4B	110.3
C3—N3—H3D	106 (4)	H4A—C4—H4B	108.5
Co1—N3—H3D	115 (4)	N5—C5—C6	107.0 (3)
H3C—N3—H3D	111 (5)	N5—C5—H5A	110.3
C4—N4—Co1	109.7 (2)	C6—C5—H5A	110.3
C4—N4—H4C	110 (3)	N5—C5—H5B	110.3
Co1—N4—H4C	106 (3)	C6—C5—H5B	110.3
C4—N4—H4D	114 (3)	H5A—C5—H5B	108.6
Co1—N4—H4D	114 (3)	N6—C6—C5	106.7 (3)
H4C—N4—H4D	102 (4)	N6—C6—H6A	110.4
C5—N5—Co1	110.6 (2)	C5—C6—H6A	110.4
C5—N5—H5C	106 (3)	N6—C6—H6B	110.4
Co1—N5—H5C	107 (3)	C5—C6—H6B	110.4
C5—N5—H5D	105 (3)	H6A—C6—H6B	108.6
			
Co1—N1—C1—C2	−39.6 (3)	N3—C3—C4—N4	−49.3 (4)
Co1—N2—C2—C1	−37.5 (4)	Co1—N5—C5—C6	36.0 (3)
N1—C1—C2—N2	49.9 (4)	Co1—N6—C6—C5	40.5 (3)
Co1—N3—C3—C4	37.5 (3)	N5—C5—C6—N6	−49.1 (4)
Co1—N4—C4—C3	38.7 (3)		

### Hydrogen-bond geometry (Å, º)

**Table:**

*D*—H···*A*	*D*—H	H···*A*	*D*···*A*	*D*—H···*A*
N1—H1*C*···I2	0.87 (5)	2.83 (5)	3.598 (3)	149 (4)
N1—H1*D*···I2^i^	0.87 (5)	2.79 (5)	3.616 (3)	158 (4)
N2—H2*C*···I3^ii^	0.89 (5)	3.04 (5)	3.823 (3)	149 (4)
N2—H2*D*···I1^iii^	0.80 (5)	3.02 (5)	3.756 (4)	154 (4)
N3—H3*C*···I2^iv^	0.97 (5)	3.25 (5)	4.111 (3)	149 (4)
N3—H3*C*···I5^iv^	0.97 (5)	3.08 (5)	3.586 (3)	114 (3)
N3—H3*D*···I1^iii^	0.76 (5)	3.18 (5)	3.805 (4)	142 (5)
N4—H4*C*···I1	0.92 (5)	2.77 (5)	3.630 (3)	155 (4)
N4—H4*D*···I1^v^	0.90 (5)	2.90 (5)	3.715 (3)	152 (4)
N5—H5*C*···I1^v^	0.89 (5)	2.95 (5)	3.765 (3)	154 (4)
N5—H5*C*···I3^ii^	0.89 (5)	3.25 (5)	3.639 (3)	109 (3)
N5—H5*D*···I4^ii^	0.89 (5)	3.05 (5)	3.611 (3)	123 (4)
N6—H6*C*···I5	0.91 (5)	2.89 (5)	3.674 (3)	145 (4)
N6—H6*D*···I2^iv^	0.95 (5)	2.80 (5)	3.684 (3)	157 (4)

Symmetry codes: (i) −*x*+1, *y*+1/2, −*z*+1/2; (ii) −*x*, *y*+1/2, −*z*+1/2; (iii) *x*+1/2, −*y*+1/2, −*z*+1; (iv) −*x*+1, *y*−1/2, −*z*+1/2; (v) *x*−1/2, −*y*+1/2, −*z*+1.
